# Increased Corneal Toricity after Long-Term Orthokeratology Lens Wear

**DOI:** 10.1155/2018/7106028

**Published:** 2018-10-23

**Authors:** Zhi Chen, Jiaqi Zhou, Feng Xue, Xingtao Zhou, Xiaomei Qu

**Affiliations:** ^1^Department of Ophthalmology and Vision Science, Eye and ENT Hospital, Fudan University, Shanghai, China; ^2^NHC Key Laboratory of Myopia, Fudan University, Shanghai, China; ^3^Laboratory of Myopia, Chinese Academy of Medical Sciences, Shanghai, China

## Abstract

**Purpose:**

To investigate the change in corneal toricity and associated refractive astigmatism after discontinuation of long-term orthokeratology (ortho-k) lens wear.

**Methods:**

This study investigated 136 subjects aged between 6 and 14 (9.1 ± 1.5) years old at the commencement of ortho-k treatment, who had been undergoing overnight ortho-k treatment for 24 to 72 (37.4 ± 11.9) months. Corneal refractive power and manifest refraction were measured and compared before ortho-k and 1 month after discontinuation of ortho-k lens wear. Changes in corneal curvature were analyzed. Corneal curvature data from a historical longitudinal study were used as control.

**Results:**

Compared to pre-ortho-k values, the corneal curvature became significantly flatter in the flatter meridian (−0.22 ± 0.27 D, *P* < 0.001) and steeper in the steeper meridian (0.06 ± 0.34 D, *P*=0.032) after cessation of ortho-k lens wear, resulting in a significant increase in corneal toricity (0.28 ± 0.43 D, *P* < 0.001), which is associated with an increase in refractive astigmatism (0.57 ± 0.57 D, *r*=0.465, *P* < 0.001). The amount of residual corneal flattening in the flatter meridian is significantly affected by the length of ortho-k treatment (*t*=−2.965, *P*=0.004) and the baseline age of subject (*t*=−2.841, *P*=0.005), but not by the baseline spherical or cylindrical refractive error (both *P* > 0.05). In the historical control group, there is no significant change in the corneal curvature over two years in children wearing spectacle lenses (both meridians, *P* > 0.05). Change of corneal toricity was more significant in the ortho-k group than in the spectacle control group (*P*=0.001).

**Conclusions:**

Long-term ortho-k lens wear increases corneal toricity after discontinuation of the treatment, which is associated with an increase in refractive astigmatism. A more pronounced change in corneal toricity was found in subjects who were younger to start ortho-k and have been in a longer period of treatment. This trial is registered with http://www.chictr.org.cn (ChiCTR-TNRC-11001210).

## 1. Introduction

Modern orthokeratology (ortho-k) is a nonsurgical procedure designed to temporarily reduce refractive error and improve uncorrected vision by the application of a reverse-geometry gas-permeable rigid contact lens [1, 2]. Ortho-k for myopic correction alters the corneal curvature that makes the central cornea flatter and midperipheral cornea steeper [3]; the mechanism underlying the corneal curvature changes has been shown to be mainly epithelium in origin [4], and these changes are believed to be reversible should patients discontinue ortho-k lens wear for a sufficient length of time [5]. However, little is known about the residual corneal curvature change after long-term ortho-k lens wear.

Barr et al. [6] were the first to investigate the recovery process following overnight reverse-geometry ortho-k lens wear and reported that 72 hours of discontinuation was insufficient for the cornea to recover. Later, Soni et al. [5] conducted a study in which the subjects underwent overnight ortho-k treatment for one month and stopped lens wear for two weeks. After the 2-week washout period, corneal curvature and refractive error largely returned to baseline. However, the treatment period is too short to draw a safe conclusion that the corneal reshaping effect can be fully eliminated after discontinuation of ortho-k lens wear. Kobayashi et al. [7] therefore carried out a 60-week clinical observation, with the first 52 weeks being the ortho-k treatment period and the last 8 weeks being the washout period. Their data showed a mild but insignificant hyperopic shift in refractive error after 8 weeks of discontinuation of ortho-k lens wear. However, they did not report the corneal curvature data.

Santodomingo-Rubido et al. [8] followed the subjects undergoing ortho-k therapy for 24 months and stopped lens wear in them for one week. They reported that the effects of long-term ortho-k on corneal curvature and refraction are still present after 1-week discontinuation of lens wear. It seems necessary to stop lens wear for a longer period to further testify the reversibility of ortho-k treatment. Credit for the understanding of this issue must be shared by Wu, Stapleton, and Swarbrick, who treated children subjects with ortho-k for an average of 50 months and discontinued lens wear for an average of 17 days [9]. Interestingly, they found a significant residual corneal flattening in the flatter meridian after the discontinuation period, which opens the possibility that long-term ortho-k lens wear has some minor permanent effect on corneal curvature. In agreement with their findings, in our clinical practice, we observed corneal flattening in the flatter meridian and a resultant increase in corneal toricity in some of our patients after discontinuation of long-term ortho-k lens wear. However, without a control group, the question remains open as whether these changes are specific to ortho-k treatment or physiological (i.e., aging effect in children free from ortho-k treatment).

The aim of this study was to investigate the change in corneal toricity and associated refractive astigmatism after discontinuation of long-term ortho-k lens wear and to compare it with children wearing spectacles from a historical control group.

## 2. Methods

### 2.1. Ortho-k Subjects

This retrospective study adhered to the tenets of the Declaration of Helsinki. In this study, 136 Chinese subjects who visited the Fudan University Eye and ENT Hospital (Shanghai, China) between August 2017 and January 2018 were consecutively included. The subjects were aged between 6 and 14 (9.1 ± 1.5) years old at the commencement of ortho-k treatment. They have been undergoing ortho-k therapy for a length of 24 to 72 months (37.4 ± 11.9 months) at the time of enrollment and were required to cease lens wear for various reasons, e.g., loss of lens, prescription updates for myopia progression, or routine checkup before reorder. The discontinuation period was 1 month in all cases. Exclusion criteria for this analysis were (1) pre-ortho-k spherical refractive error greater than −5.00 DS (based on noncycloplegic manifest refraction) and (2) limbus-to-limbus corneal astigmatism in the need of toric designed ortho-k lenses as a treatment (a toric designed lens is supposed to yield different tensions on the steeper meridian than a spherical lens does and might confound the corneal curvature results). Ortho-k was performed in both eyes of the subjects but only data from the right eyes were analyzed.

### 2.2. Ortho-k Lenses

The ortho-k contact lenses worn by all the subjects were spherical four-zone reverse geometry gas-permeable rigid contact lenses (Emerald Series, Euclid, USA) composed of oprifocon A (Boston Equalens II). The lens has a back optical zone diameter (BOZD) of 6.2 mm, a reverse curve of 0.5 mm width, an alignment curve of 1.2 mm width, and a peripheral curve of 0.5 mm width. The total diameter of a typical trial lens is 10.6 mm, and the central thickness is 0.22 mm. Final lenses were prescribed with a total diameter (TD) tailored to the horizontal visible iris diameter (HVID) using the following equation (the maximum TD that can be ordered was 11.4):

(1) $$\begin{array}{c} \mathrm{TD}=\mathrm{HVID}-1.0\pm 0.1\;\mathrm{mm}. \end{array}$$

Over-refraction was performed before the final lenses were ordered. A Jessen factor of 0.75 D was used in all cases. Should significant lens decentration (greater than 1.0 mm) occur or the unaided visual acuity drop below 20/25 during follow-up visits, new lenses would be ordered until a good lens centration was regained and visual acuity restored to over 20/25; otherwise, the lenses were replaced every 12 to 18 months on a regular basis.

### 2.3. Corneal Topography

Corneal topography was measured with the Placido ring-based Medmont topographer (E300, Medmont, Australia), prior to ortho-k treatment and at every follow-up visit, including the end of the discontinuation period. At least two measurements were taken with difference in K readings not greater than 0.05 D along either meridian. Only those topography maps with an optimal quality (no significant rhinal shade or tear film breakup) were included in the final analysis. The corneal curvature as expressed by simulated K in diopters was recorded before ortho-k treatment and after discontinuation of lens wear.

### 2.4. Control Group

The control data were from 123 spectacle-wearing children who were enrolled in our earlier studies (data unpublished). They were aged between 6 and 14 (9.4 ± 1.8) years old at baseline and completed the 2-year follow-up study. Corneal topography using Medmont topographer was performed both before treatment and at the completion of follow-up visits. Criteria for the selection of data were identical to the ortho-k group.

### 2.5. Data Analysis

Refractive sphere and cylinder, corneal curvatures along both meridians, and corneal toricity were compared before ortho-k and after discontinuation of ortho-k lens wear using the paired samples *t*-test. The effects of pre-ortho-k refractive error, age, and duration of ortho-k treatment on the change in corneal curvature were analyzed using stepwise multiple linear regression analysis. The relationship between the change of corneal toricity and refractive cylinder was analyzed using the Pearson correlation test. Refractive error and corneal curvatures of the subjects were compared between the ortho-k group and the spectacle control group prior to and after treatment using independent samples *t*-test. A *P* < 0.05 was considered to be statistically significant.

## 3. Results

Spherical and cylindrical refractive error was −2.53 ± 1.07 DS (range, −4.75 to −1.00 DS) and −0.31 ± 0.40 DC (range, −1.50 to 0 DC) before ortho-k treatment and −3.39 ± 1.04 DS (range, −5.50 to −1.00 DS) and −0.88 ± 0.50 DC (range, −2.00 to 0 DC) after 37.4 ± 11.9 months of ortho-k treatment followed by one month of lens wear discontinuation, with both changes being statistically significant (both *P* < 0.001).

**Figure 1:**
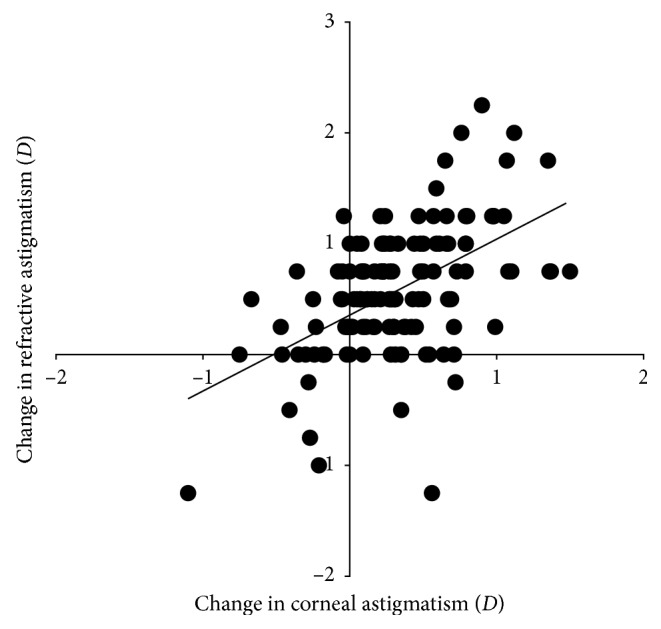
Scatterplots showing the correlation between the change in corneal and refractive astigmatism after discontinuation of ortho-k lens wear as compared to pre-ortho-k value.

Corneal curvature along the flatter and steeper meridian changed from 42.84 ± 0.98 D (range, 40.48 to 45.26 D) and 44.00 ± 1.17 D (41.00 to 46.46 D) before ortho-k treatment to 42.62 ± 1.01 D (40.02 to 45.01 D) and 44.06 ± 1.11 D (41.02 to 46.99 D) after discontinuation of ortho-k lens wear, respectively. Compared to baseline, corneal curvature became significantly flatter in the flatter meridian (−0.22 ± 0.27 D, *P* < 0.001) and steeper in the steeper meridian (0.06 ± 0.34 D, *P*=0.032) after cessation of ortho-k lens wear, resulting in a significant increase in with-the-rule corneal astigmatism (0.28 ± 0.43 D, range −1.23 to 1.50 D, *P* < 0.001), which is associated with an increase in refractive astigmatism (0.57 ± 0.57 D, *r*=0.523, *P* < 0.001; Figure 1).

**Figure 2:**
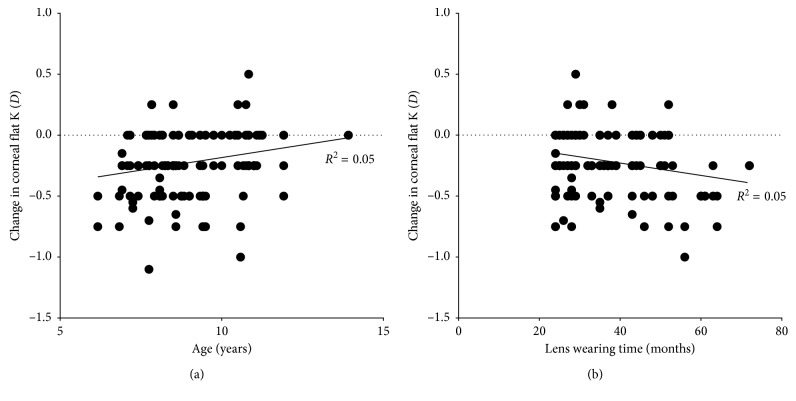
Scatterplots showing the change in corneal flat K after cessation of ortho-k lens wear, in the function of subject's starting age for ortho-k (a) and lens wearing time (b).

The amount of residual corneal flattening in the flatter meridian is significantly affected by the length of ortho-k treatment (*t*=−2.965, *P*=0.004) and the starting age of ortho-k treatment (*t*=2.841, *P*=0.005), but not by spherical or cylindrical refractive error prior to ortho-k (both *P* > 0.05). Younger subjects and those who have had longer ortho-k treatment experienced greater increase in corneal astigmatism after discontinuation of lens wear (Figure 2).

**Figure 3:**
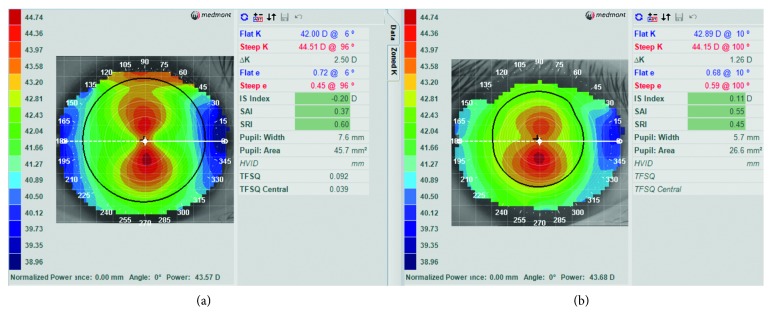
Corneal topography maps from a representative subject who experienced a significant increase in corneal toricity and a change in overall corneal shape in the right eye after discontinuation of ortho-k lens wear (a) as compared to pre-ortho-k value (b).

After 1-month discontinuation of ortho-k lens wear, all the 136 subject eyes were refitted with ortho-k lenses, among which 25 switched from original spherical designs to toric designs.  Figure 3 shows corneal topography maps before ortho-k treatment and after discontinuation of 2 years lens wear in a representative subject who experienced an increase in corneal toricity and a change in the overall corneal shape.

Before treatment, age, spherical refractive error, flat K, and steep K were similar between the ortho-k group and the historical control group, while cylindrical refractive error and corneal toricity were slightly higher in the control group than in the ortho-k group (Table 1). Over the 2-year course of treatment, the corneal curvature did not significantly change in children wearing spectacle lenses (both meridians, *P* > 0.05). Therefore, the change in corneal toricity was more significant in the ortho-k group (0.28 ± 0.43 D) than in the control group (0.12 ± 0.38 D; *P*=0.001).

## 4. Discussion

In this study, we found that after long-term ortho-k treatment, the corneal curvature did not fully recover to the baseline level and corneal toricity increased after 1-month discontinuation of ortho-k lens wear. The increase in corneal toricity was associated with an increase in refractive astigmatism.

By treating patients with traditional flat-fitting ortho-k lenses, Kerns [10] reported a significant induction of corneal toricity after prolonged lens wear because the corneal curvature along the steeper meridian became steeper than baseline after lens discontinuation. Increased corneal toricity associated with traditional ortho-k lens wear was mainly caused by significant lens decentration and confounded by low oxygen-permeable lens material related hypoxic corneal effects. In modern orthokeratology, most of the ortho-k lenses being fitted are of high oxygen permeability, well centered, and are supposed to yield relatively even forces to different corneal meridians. Nevertheless, we found a significant change in the corneal curvature after discontinuation of lens wear following long-term ortho-k treatment, despite that a good lens centration had been achieved using reverse-geometry lenses.

While a mean change of 0.28 D in corneal toricity may seem clinically insignificant, noteworthy is that 25 out of 136 subjects had to be refitted ortho-k lenses with midperipheral toric designs after the washout period as opposed to original spherical designs, indicating that their overall anterior corneal shape has been changed in addition to central curvature change. In agreement with our study, Wu et al. [9] found the persistence of a small increase of with-the-rule corneal astigmatism (0.17 D) after discontinuation of reverse geometry ortho-k lens wear, due to a residual corneal flattening in the flatter meridian. The authors also found a trend toward greater residual corneal flattening among subjects with higher pretreatment myopic refractive error. However, the discontinuation period in their study ranged from 7 to 45 days (mean, 17 days), which might be inadequate for the cornea to recover in some of the subjects with shorter period of washout, as indicated by Santodomingo-Rubido et al.'s study [8]. Our study did not find a significant correlation between initial refractive error and residual corneal flattening after a uniform 1-month lens discontinuation, suggesting that ortho-k wearers with higher myopia do not necessarily have to stop lens wear for longer periods for their corneas to recover as compared to their lower myopic counterparts.

Interestingly, our results indicated that age might be an influencing factor for corneal recovery after long-term ortho-k lens wear, although it only explains 5% of the variability. A previous study reported that older subjects (43.9 ± 6.1 years) experienced delayed response to short-term myopic ortho-k treatment. After one hour of lens wear, visual acuity, refraction, and corneal topography changes were significantly less compared to children (9.5 ± 1.7 years) and young adult subjects (24.6 ± 3.7 years) [11]. However, to the best of our knowledge, no studies have investigated the effect of age on corneal biomechanical properties among children aged between 6 and 14 years, but it is possible that physiological changes in a patient's cornea may occur during long term of ortho-k lens wear, especially in younger children.

Another possible explanation for the incomplete corneal recovery was insufficient lens discontinuation. Kang and Swarbrick [12] recently reported a case, in which a Caucasian female continuously wore ortho-k lenses for 13 years, stopped lens wear for 408 days, and then went for refractive surgery. During the 408 days washout period, the authors found that her corneal curvature almost returned to pre-ortho-k value (about 0.25 D flatter) in one month but did not completely recover until more than one year later. It could be argued that 1-month lens discontinuation might be inadequate in the current study, but it is unlikely for some of the corneas that experienced greater than 1 D increase in corneal toricity to fully recover over time (Figure 2; we actually continued to observe these subjects further but failed to see a significant change even after three months).

Since a more significant residual corneal flattening was observed in the subjects who underwent longer period of ortho-k treatment (Figure 2), it could be argued that the corneal curvature change in children was merely due to an aging effect rather than ortho-k treatment per se. However, data from the spectacle control group are not supportive of this notion: flat K, steep K, and corneal toricity did not significantly change over time in the control group. But given that the observational period was only two years for the control group, as opposed to as long as six years in the ortho-k group, it does not negate the possibility of corneal toricity change in the longer term even in the absence of ortho-k treatment, which warrants further investigation.

It should be noted that all the lenses used in this study were spherical in design, as no limbus-to-limbus corneal astigmatism was present at baseline and spherical lenses have achieved full-correction and good lens centration in all cases. Even if most of the spherical ortho-k lenses were fitted seemingly in alignment with the midperipheral cornea along all the meridians, they could have caused uneven compressive stress on different meridians, being greater in the flatter (horizontal) meridian than in the steeper (vertical) meridian, which in the long term may cause disparate change in corneal physiology between the two meridians. As a result, when being refitted with ortho-k lenses after the discontinuation period, 25 out of 136 subject eyes had a significant increase in corneal toricity and thus needed toric-designed lenses to maintain good lens centration and visual correction. It would be interesting to investigate the long-term effect of toric ortho-k lens wear on the corneal curvature as toric lenses are supposed to induce a more uniform pressure to the midperipheral cornea along different meridians when significant corneal toricity is available.

Yang et al. [13] investigated the corneal curvature change in subjects after 24 to 96 months ortho-k treatment followed by 1 to 36 months discontinuation of lens wear, using a mixture of spherical and toric-designed ortho-k lenses. Although they reported no significant change in the corneal curvature after 3 months discontinuation of lens wear as compared to baseline, they did not compare between spherical lens design and toric lens design. Further studies are warranted to investigate the effect of different lens designs on long-term corneal curvature.

In conclusion, this study revealed a significant increase in corneal toricity after long-term ortho-k treatment, and the increase in corneal toricity was associated with an increase in refractive astigmatism. A more pronounced change in corneal toricity was found in subjects who were younger to start ortho-k and have been in a longer period of treatment.

## Figures and Tables

**Table 1:** Comparison of refractive error and corneal curvature between the ortho-k and control group.

	OK (*n*=136)		SVL (*n*=123)		*P* value
	Mean	SD	Mean	SD	
Pretreatment age	9.1	1.5	9.4	1.8	0.149
Pretreatment sphere	−2.53	1.07	−2.37	1.52	0.336
Pretreatment cylinder	−0.31	0.40	−0.45	0.58	0.024
Pretreatment FK	42.84	0.98	42.84	1.34	0.970
Pretreatment SK	44.00	1.17	44.11	1.57	0.494
Pretreatment toricity	1.16	0.41	1.28	0.57	0.047
Posttreatment FK	42.62	1.01	42.74	1.35	0.430
Posttreatment SK	44.06	1.12	44.14	1.58	0.645
Posttreatment toricity	1.44	0.44	1.40	0.61	0.556
Change in toricity	0.28	0.43	0.12	0.38	0.001

## Data Availability

The spreadsheet data used to support the findings of this study are included within the supplementary information files.

## Supplementary Materials

Supplementary Materials The spreadsheet data contain the baseline refractive error and corneal curvature, the starting age and length of ortho-k treatment, refractive error, and corneal curvature at the end of ortho-k treatment, after cessation of lens wear.
