# Supplementary material for: Increased Corneal Toricity after Long-Term Orthokeratology Lens Wear
Source: J Ophthalmol. 2018 Oct 23;2018:7106028. doi: 10.1155/2018/7106028 (PMC6218724; doi:10.1155/2018/7106028)
Supplement: Supplementary Materials — The spreadsheet data contain the baseline refractive error and corneal curvature, the starting age and length of ortho-k treatment, refractive error, and corneal curvature at the end of ortho-k treatment, after cessation of lens wear. [file 7106028.f1.pdf]

BL Sph (D)	BL Cyl (D)	BL Age (y)	LW Time (m)	Final Sph (D)	Final Cyl (D)	BL FK (D)	Final FK (D)	BL SK (D)	Final SK (D)
−1.75	0	10.83	72	-4.25	-1.25	41.98	41.74	42.52	42.74
−1	−0.5	10.83	72	-2.75	-0.75	41.98	41.74	43.27	43.24
−1.75	0	9.42	64	-2.75	-1.25	44.48	43.74	45.52	45.74
−1.5	0	9.42	64	-2	-1	43.98	43.49	45.27	45.24
−2.5	0	8.17	63	-3.5	0	40.48	39.99	41.02	41.24
−2.75	0	8.17	63	-3.5	0	40.48	40.24	41.27	40.99
−2.75	−0.5	9	61	-3.5	-1	41.98	41.49	43.27	42.99
−1.5	−0.75	9	61	-3.75	-1.25	41.98	41.49	43.52	42.99
−3.25	0	8.08	60	-5.25	-1.25	42.73	42.24	43.27	43.24
−2.75	−0.5	8.08	60	-4.5	-1.5	42.48	41.99	43.27	42.99
−2.5	−0.75	10.58	56	-2.5	-2	43.23	42.24	44.77	44.74
−1.5	−1.25	10.58	56	-2	-1.75	43.48	42.74	45.02	44.74
−1.5	−0.5	8.83	53	-2.25	-1.5	42.98	42.49	44.02	44.24
−1.5	−0.5	8.83	53	-2.75	-1.5	42.73	42.49	43.77	43.99
−2	0	9.5	52	-2.25	-1.25	45.23	44.47	46.52	46.97
−2.25	0	9.5	52	-2	-0.75	44.73	44.24	46.27	46.74
−1.25	−1	11.92	52	-3.75	-2	42.98	42.99	44.02	44.74
−2.25	−1.25	11.92	52	-3.5	-1.75	42.73	42.74	44.02	44.49
−1.5	−0.5	10.75	52	-2	-1.75	42.48	42.72	43.77	44.22
−4	−0.5	10.75	52	-2	-1	42.98	42.97	44.02	43.97
−2.25	0	7.08	51	-4	-0.5	43.48	43.22	44.52	43.97
−1.25	−1.75	7.08	51	-3	-0.5	42.73	42.72	45.02	43.78
−2	0	7.75	50	-4.25	-0.75	41.23	40.97	42.02	42.72
−3.75	−1.5	10.75	50	-4.25	-0.5	41.48	41.22	43.27	43.47
−3.5	0	10.75	50	-4	-0.25	41.98	41.97	42.77	42.97
−2	0	7.75	50	-4	-1	41.23	40.97	42.02	41.72
−3.5	−0.5	8.75	48	-4.25	-0.75	40.98	40.47	42.02	42.22
−3.25	−0.25	8.75	48	-3.75	-1.25	40.98	40.47	41.52	41.22
−1.75	−0.5	9.5	48	-3.5	-0.75	44.23	44.22	45.27	45.22
−0.75	−1	9.5	48	-2.5	-1	43.48	43.47	45.27	45.22

−1. 5	0	6. 17	46	-3	-1.75	43. 48	42. 72	44. 52	44. 72
−1. 25	0	6. 17	46	-2	-1.75	43. 23	42. 72	44. 27	44. 22
−1. 5	0	7. 17	45	-3.5	-0.75	43. 23	43. 22	44. 02	44. 72
−3. 25	−0. 75	9. 33	45	-2.75	-1	42. 47	42. 49	43. 68	43. 99
−3	−0. 75	9. 33	45	-2.25	-0.5	42. 22	41. 99	43. 18	43. 24
−1. 5	0	7. 17	45	-2	-1.25	43. 48	43. 47	44. 77	44. 97
−2	0	8. 67	45	-4.25	-1.25	42. 97	42. 99	43. 68	43. 74
−2	−0. 5	8. 67	45	-4	-1.5	43. 22	43. 24	44. 43	44. 49
−1. 75	0	8. 5	44	-2.5	-1	40. 72	40. 49	41. 43	42. 49
−1. 75	0	8. 5	44	-2	-0.75	40. 47	40. 49	41. 18	41. 74
−2	−0. 5	7. 67	44	-3.75	0	42. 72	42. 74	43. 68	43. 74
−1. 75	−0. 5	7. 67	44	-3.25	-0.75	41. 97	41. 99	43. 43	43. 49
−1. 25	0	8. 58	43	-2	-0.75	41. 72	41. 49	42. 43	43. 49
−1. 5	0	8. 58	43	-2.5	-1.25	42. 22	41. 59	42. 93	43. 39
−2	0	7. 92	43	-2.75	0	43. 47	43. 24	43. 93	44. 24
−2. 25	0	7. 92	43	-2.5	-0.5	43. 97	43. 49	44. 68	44. 74
−1. 75	−0. 75	13. 92	43	-5.25	-1	43. 72	43. 74	44. 68	44. 99
−4. 75	−0. 5	13. 92	43	-4	-1	43. 47	43. 49	45. 18	44. 74
−2	−0. 5	8. 25	39	-3.25	-1.5	43. 72	43. 49	45. 18	45. 49
−1. 75	−0. 75	8. 25	39	-3	-1.5	43. 97	43. 74	45. 43	45. 49
−1	−0. 5	11. 25	39	-1.75	0	43. 47	43. 49	44. 93	44. 74
−0. 75	−0. 5	11. 25	39	-1.5	-0.5	43. 72	43. 69	45. 18	44. 94
−2. 5	−0. 5	8. 5	38	-3.75	-0.25	42. 22	41. 94	43. 18	42. 94
−2. 5	0	8. 5	38	-4	-0.75	41. 97	42. 19	43. 18	42. 94
−2. 25	0	6. 92	37	-3.25	-1.25	42. 97	42. 69	43. 18	43. 69
−2	0	9. 42	37	-3.25	-1	42. 47	41. 94	43. 68	43. 69
−2	0	9. 42	37	-3.5	-0.5	42. 47	42. 19	43. 43	43. 69
−1. 5	−0. 5	7. 17	37	-3.75	-0.5	42. 22	41. 69	43. 43	43. 44
−2. 25	0	11. 17	37	-2.75	-0.75	42. 47	42. 44	43. 18	43. 69
−2. 5	−1. 25	11. 17	37	-2.5	-0.5	42. 47	42. 44	43. 43	43. 94
−2	0	6. 92	37	-2.75	-1	42. 97	42. 69	43. 43	43. 44

−2.5	−0.5	7.17	37	-2.75	-1.5	41.97	41.69	43.18	43.19
−3.5	0	8.33	36	-4	0	43.72	43.44	45.43	44.69
−3.75	−0.5	8.33	36	-3.75	-0.5	43.47	43.19	45.43	44.44
−3.25	−0.25	7.25	35	-4.25	-0.5	43.47	42.84	44.18	44.04
−3.25	0	7.25	35	-4	0	43.22	42.64	43.93	43.74
−3	0	9	35	-3	-0.5	43.52	43.54	44.04	44.04
−2	0	9	35	-2	-0.75	42.77	42.79	43.54	43.54
−1.5	0	8.17	35	-3.5	-0.75	44.47	44.44	45.68	45.44
−3	−0.75	12.5	35	-3.5	-1.25	44.27	44.05	46.04	45.55
−2.75	−1	8.5	35	-5	-0.5	44.52	44.29	46.29	45.79
−1.75	0	8.17	35	-4	-1	44.47	44.44	45.93	45.44
−4	0	10.67	33	-4	-0.5	41.77	41.29	42.79	42.79
−2.75	0	7.42	33	-3.25	-1	43.77	43.54	45.04	45.04
−3.25	0	10.67	33	-3.25	-0.5	42.02	41.79	43.04	43.04
−2.5	0	7.42	33	-4	-0.75	43.77	43.54	45.04	44.79
−3.25	−0.5	11	32	-5	-0.75	43.52	43.29	44.79	45.04
−4	−1	11	32	-5	-1	42.77	42.54	44.29	43.54
−2.5	0	10.5	31	-4.5	-0.5	43.52	43.79	44.79	45.04
−3	−0.5	10.5	31	-4.5	-0.75	43.52	43.54	45.29	45.29
−3.5	−0.75	7.83	30	-5	-0.75	42.52	42.54	44.29	44.54
−4.25	0	10.25	30	-5	-1.25	43.27	43.29	44.54	44.54
−4.5	−0.5	10.25	30	-4.5	-1	43.27	43.29	45.04	44.79
−3.5	−1	7.83	30	-4.5	-1	42.52	42.79	44.54	44.54
−3.25	0	11.92	29	-5.25	-0.5	42.27	41.79	43.29	43.54
−4.5	−0.5	9.75	29	-5.25	-0.5	42.27	42.29	43.79	44.04
−3.5	0	11.92	29	-3.75	-0.5	42.77	42.54	43.54	43.54
−3.25	−0.5	9.75	29	-3.25	-1	41.52	41.5	42.79	43
−4.75	−0.75	10.83	29	-2	-0.75	44.77	44.75	45.79	46
−4.75	−0.5	9.75	29	-4.25	-1	42.27	42.04	44.04	43.79
−1.5	−0.5	9.75	29	-4.5	-0.75	41.27	41.25	42.79	42.75
−4.75	−1	10.83	29	-4.75	-1	44.27	44.75	45.79	46

-1.5	0	8.58	28	-1.75	-0.5	43.77	42.98	44.54	45.25
-1	-0.75	7.42	28	-2.25	-0.5	43.27	42.75	45.04	45
-1.25	0	8.08	28	-1.5	-2	42.27	41.8	43.04	42.9
-2	0	8.08	28	-1.5	-1.75	42.27	41.9	43.29	43.2
-2.25	-0.5	7.42	28	-4.5	-0.25	43.27	43	45.04	45
-2	0	8.58	28	-3.25	-0.75	43.77	43.5	44.54	44.25
-1.25	-0.25	10.42	28	-1.75	-1.25	43.27	43.25	45.04	45
-1.5	-0.25	10.42	28	-1.25	-1	43.52	43.5	44.79	44.75
-1.25	-0.75	9.33	27	-4.25	-1	44.53	44.01	45.55	46.01
-2	0	7.67	27	-3.25	-1	41.77	41.5	43.04	43.25
-1	0	7.83	27	-3	-0.5	42.02	42	42.54	43
-1.25	0	7.83	27	-3.25	-0.5	42.03	42.26	42.55	43.26
-2	0	9.33	27	-3.25	0	45.03	45.01	45.8	46.26
-3	-0.75	10.5	27	-2	-1	43.03	43.04	44.26	44.54
-3	-0.5	10.5	27	-3.5	-1	43.53	43.29	44.51	44.54
-2.75	0	7.67	27	-3.75	-0.75	41.77	41.75	43.04	43.25
-4.5	-0.5	11.08	26	-4.5	-1.5	43.53	42.44	45.01	45.04
-3.75	0.75	7.75	26	-4.5	-1.5	43.53	42.84	45.01	45.24
-3	0	7.75	26	-4.5	-0.5	42.53	42.29	44.01	44.04
-4.75	-0.75	9.08	26	-4.5	-1.25	42.03	42.04	43.01	43.04
-4.75	-1	9.08	26	-4	0	44.03	44.04	45.51	45.54
-4.25	-0.75	11.08	26	-3.75	-0.5	43.03	43.04	44.76	44.54
-3.25	0	7.75	26	-5	0	42.28	42.29	43.01	42.79
-4	-0.5	9.1	26	-5	0	41.78	41.77	43.26	43.02
-3.75	0	10	25	-3.5	-0.75	41.28	41.04	41.76	42.29
-2.75	0	8.67	25	-3	-0.75	43.28	43.04	44.76	44.79
-4	0	11	25	-1	-1.25	41.28	41.29	42.01	42.29
-2	0	8.67	25	-3.25	-0.75	44.03	44.04	45.76	45.79
-1	0	10	25	-4.25	-1	43.53	43.54	45.01	45.04
-3.75	0	11	25	-4.25	-1.25	43.28	43.04	44.76	44.54
-2.5	0	7.92	24	-3.75	-1.75	42.28	41.52	43.26	43.52

−3.25	−0.75	8.5	24	-4.5	-1.25	43.28	42.77	44.26	44.52
−4.75	0	10.83	24	-1.5	0.5	42.78	42.27	44.01	44.27
−1.25	0	6.92	24	-3	-0.25	43.53	43.27	44.51	44.77
−3.5	0	6.83	24	-3.5	-2	42.28	41.77	43.51	43.52
−2	−0.75	9.42	24	-3	-1.25	43.03	42.27	44.51	44.27
−4.75	0	10.83	24	-2.5	-1	42.28	42.02	43.26	43.52
−1.5	0	6.92	24	-2.25	-1	42.28	41.82	43.01	42.92
−2.75	0	6.83	24	-2	-0.75	42.28	42.12	42.51	42.62
−1.5	0	8.08	24	-2.25	-0.5	41.53	41.52	42.76	43.02
−2	0	9.42	24	-3	-1.5	41.53	41.52	42.76	43.02
−3.25	−0.5	8.5	24	-3.25	-1.5	43.28	43.04	45.01	44.79
−2	0	7.92	24	-5.25	-0.75	43.28	43.27	44.26	44.27
−1.25	0	8.08	24	-5.5	-0.75	43.03	43.02	43.76	43.77
